# Treatment of Scabies

**Published:** 1866-03

**Authors:** 


*Treatment of Scabies.*—From the "Medical Mirror" we learn that Dr. J. F. Nicholls, Surgeon to the Royal Wilts Militia, proposes the following plan of treating itch. He has tried it in several hundred cases, and has compared it with the plan of treatment adopted in other regiments, and finds it greatly superior to anything suggested. He declares that where the remedy is properly prepared and used it invariably effects a cure. Dr. Nicholls says:

"The remedy which I have adopted is prepared by boiling one part of quick-lime with two parts of sublimed sulphur, in ten parts of water, until the lime and sulphur are perfectly united; during the boiling it must be constantly stirred with a piece of wood, and when the sulphur and lime have combined the fluid is to be decanted and kept in a well stoppered bottle.

The way in which I have it applied is as follows: Every part of the body, with the exception of the face and scalp, is well rubbed with the preparation for half an hour, and then washed with soap and hot water. After the application an entire change of clean linen and clothing is put on. The patient's linen is disinfected by being soaked in boiling water, and his clothes are freed from contagion by being subjected to a temperature of about 200 deg. Fahr., in an oven for a short time. When using the remedy for young children I have had it diluted by mixing one part of the solution prepared as above with three parts of water."

From the Glasgow Medical Journal we learn that Dr. De Casine, physician to the garrison at Antwerp, proposes the *oil of petroleum* as an almost instantaneous mode of cure of Scabies in man. It not only kills instantaneously the parasite, but acts as a disinfectant against the larvæ found in the clothing. Dr. DeCasine has addressed a communication to the Belgian Academy of Medicine upon this mode of treatment.

